# Ontogeny of Sex-Related Differences in Foetal Developmental Features, Lipid Availability and Fatty Acid Composition

**DOI:** 10.3390/ijms18061171

**Published:** 2017-05-31

**Authors:** Consolacion Garcia-Contreras, Marta Vazquez-Gomez, Susana Astiz, Laura Torres-Rovira, Raul Sanchez-Sanchez, Ernesto Gomez-Fidalgo, Jorge Gonzalez, Beatriz Isabel, Ana Rey, Cristina Ovilo, Antonio Gonzalez-Bulnes

**Affiliations:** 1 Subdirección General de Investigación y Tecnología, Instituto Nacional de Investigación y Tecnología Agraria y Alimentaria, Madrid 28040, Spain; consolacion@inia.es (C.G.-C.); astiz.susana@inia.es (S.A.); torres.laura@inia.es (L.T.-R.); raulss@inia.es (R.S.-S.); fidalgo@inia.es (E.G.-F.); ovilo@inia.es (C.O.); 2 Faculty of Veterinary Sciences, Universidad Complutense de Madrid, Madrid 28040, Spain; mvgomez@ucm.es (M.V.-G.); bisabelr@ucm.es (B.I.); anarey@ucm.es (A.R.); 3 Micros Veterinaria, Leon 24007, Spain; info@microsvet.com

**Keywords:** fatty-acids, foetus, lipids, metabolism, nutrition, sex

## Abstract

Sex-related differences in lipid availability and fatty acid composition during swine foetal development were investigated. Plasma cholesterol and triglyceride concentrations in the mother were strongly related to the adequacy or inadequacy of foetal development and concomitant activation of protective growth in some organs (brain, heart, liver and spleen). Cholesterol and triglyceride availability was similar in male and female offspring, but female foetuses showed evidence of higher placental transfer of essential fatty acids and synthesis of non-essential fatty acids in muscle and liver. These sex-related differences affected primarily the neutral lipid fraction (triglycerides), which may lead to sex-related postnatal differences in energy partitioning. These results illustrate the strong influence of the maternal lipid profile on foetal development and homeorhesis, and they confirm and extend previous reports that female offspring show better adaptive responses to maternal malnutrition than male offspring. These findings may help guide dietary interventions to ensure adequate fatty acid availability for postnatal development.

## 1. Introduction

Prenatal development of humans and animals requires adequate placental supply of oxygen and nutrients [1,2], which, in turn, requires adequate maternal nutrition and placental function. Inadequate placental nutrient supply leads to intrauterine growth restriction (IUGR), resulting in newborns that are small for their gestational age. Such offspring may be predisposed to perinatal morbidity and mortality [3] as well as lifelong chronic non-communicable disorders such as obesity, type II diabetes, hypertension and cardiovascular diseases [4,5,6,7].

Among humans, IUGR due to maternal malnutrition shows a much higher incidence in resource-challenged areas (15%) than in developed areas (6%) [8,9]. In recent years, incidence has been increasing in developed countries because of maternal eating disorders, voluntary intake restriction for aesthetic reasons [10] and abnormal placental development leading to placental insufficiency [11]. This abnormal placental development has been associated with postponement of childbearing, inadequate lifestyle and maternal and gestational factors [8,12,13].

Previous studies from our group using a swine model of IUGR have highlighted the importance of maternal nutrition for proper foetal development. Maternal malnutrition compromises foetal metabolism and development, causing growth retardation and triggering adaptive changes in the foetus to increase likelihood of survival [14]. Work from other groups has shown that the maternal lipid profile and placental efficiency are strongly related to cholesterol and triglyceride availability for human and other animal foetuses, which affects their viability and growth [15,16,17,18,19,20]. The developing foetus requires substantial amounts of lipids, mainly polyunsaturated fatty acids (PUFA), the availability of which is determined largely by maternal circulating levels and placental transfer [21]. Impaired placental transfer of PUFA causes or exacerbates IUGR [21,22,23] and may be caused by lipid peroxidation or other forms of oxidative stress [24,25].

Extensive research in humans and animal models has shown that the sex of the offspring can affect pre- and postnatal development in compromised pregnancies such as IUGR [14,26,27,28,29,30,31]. Less clear is whether this sex effect extends to lipid homeorhesis. Therefore the present study exploited the power of the pig as a translational model [32] to investigate sex-related differences in lipid availability and fatty acid composition during foetal development. The pig, like humans, is omnivorous and prone to sedentary behaviour and obesity, and the metabolism, cardiovascular system and relative organ sizes in pigs are similar to those in humans [33,34,35,36]. A further advantage of studying swine is that insights will impact not only research but also farm productivity. IUGR occurs often in swine because of inadequate maternal nutrition or placental insufficiency [2,37,38], and it limits farm profitability [14].

## 2. Results

Foetuses were obtained from five sows on Gestational Day (GD) 70 (*n* = 33) or from four sows on GD 90 (*n* = 23). The average number of foetuses per sow was 6.8 ± 3.8 on GD 70 and 5.8 ± 3.6 on GD 90. Of the 56 foetuses, 23 were female, of which 13 were recovered on GD 70 and 10 on GD 90; among the 33 male foetuses, 20 were recovered on GD 70 and 13 on GD 90. Hence, the sex ratio was close to 1:1, with males accounting for 57.7% of GD 70 foetuses and 56.5% of GD 90 foetuses. Evidence of IUGR was found in eight pregnancies, affecting four GD 70 foetuses (11%) and four GD 90 foetuses (17.4%).

### 2.1. Effects of Maternal Metabolic Status on Foetal Development

The size and weight of normal (non-IUGR) foetuses increased with gestational age (Table 1), as did development of the placenta, liver, kidney and intestine, based on histology (all *p* < 0.0001). 

**Figure 1:**
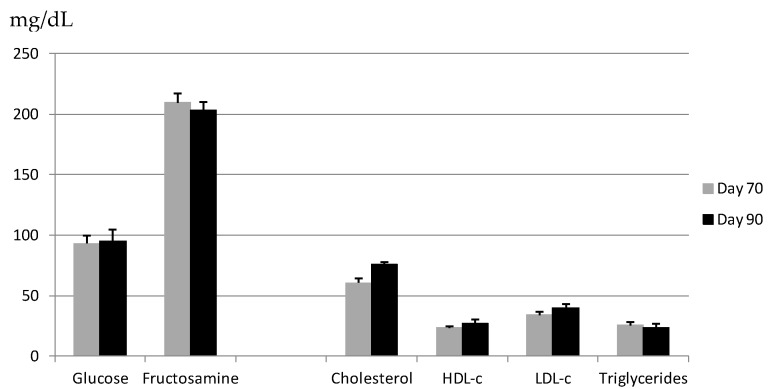
Metabolic profiles of sows, from which foetuses were harvested on Gestational Day (GD) 70 or 90. Mean fasting values (mg/dL) (±SEM) of glucose, fructosamine, total cholesterol, high-density and low-density lipoprotein-cholesterol (HDL-c and LDL-c, respectively) and triglycerides in maternal plasma on the day of harvest.

In these normal foetuses, body development correlated strongly with maternal lipid profile depicted in Figure 1. Specifically, lower maternal plasma cholesterol concentrations correlated with lower foetal body weight (*r* = 0.777, *p* < 0.05) and corpulence in terms of trunk length (*r* = 0.832), thoracic circumference (*r* = 0.808) and abdominal circumference (*r* = 0.958) (all *p* < 0.01). Lower maternal plasma cholesterol concentrations also correlated with higher ratios of organ-to-body weight for brain (*r* = −0.774), heart (*r* = −0.752), liver (*r* = −0.679), and spleen (*r* = −0.712) (all *p* < 0.01); these correlations reflect changes to improve foetal viability. Conversely, lower maternal plasma concentrations of high-density lipoprotein cholesterol (HDL-c) correlated with lower ratios of heart-to-body weight (*r* = 0.677, *p* < 0.05). Adaptive foetal growth mechanisms were observed already on GD 70, when lower maternal plasma cholesterol concentrations correlated with higher ratios of head-to-body weight (*r* = −0.987, *p* < 0.005) and heart-to-body weight (*r* = −0.883, *p* < 0.05). At the same time, maternal HDL-c concentrations correlated with higher ratios of brain-to-body weight (*r* = −0.923) and spleen-to-body weight (*r* = −0.891, both *p* < 0.05). 

Maternal plasma concentrations of glucose and fructosamine did not affect weight of normal foetuses on GD 70, but they did show an influence on GD 90. Lower plasma fructosamine concentrations correlated with lower foetal body weight (*r* = 0.964) as well as higher ratios of head-to-body weight (*r* = −0.962) and brain-to-head weight (*r* = −0.969, all *p* < 0.05). Similarly, lower maternal glucose levels correlated with higher ratios of head-to-body weight (*r* = −0.959) and brain-to-head weight (*r* = −0.963, both *p* < 0.05). 

### 2.2. Effects of Foetal Sex on Developmental Trajectories during Pregnancy

No significant association was observed between offspring sex and foetal weight in normal foetuses (Table 1), although among male foetuses collected on GD 90, we observed a trend toward higher total body weight (*p* = 0.09) and carcass weight (*p* = 0.06) compared to female foetuses. Fat content of the carcass, the *longissimus dorsi* muscle or the liver did not vary significantly as a function of foetal sex or gestational age (Table 2).

**Figure 2:**
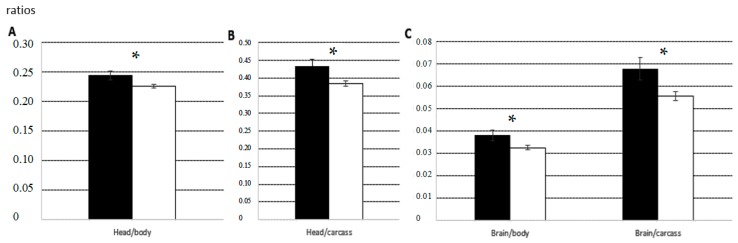
Mean ratios (±SEM) of head-to-body weight (**A**); head-to-carcass weight (**B**); and brain-to-body weight and brain-to-carcass weight (**C**) in female foetuses (black bars) and male foetuses (white bars) on Gestational Day (GD) 90. Asterisks indicate significant differences between males and females. * *p* < 0.05.

Male and female normal foetuses collected on GD 70 did not differ significantly in the relative weights or maturation states of several structures and organs. On GD 90, normal female foetuses showed a significantly higher degree of placental development (*p* < 0.05) and significantly higher ratios of head-to-body weight and brain-to-body weight (both *p* < 0.05; Figure 2).

**Figure 3:**
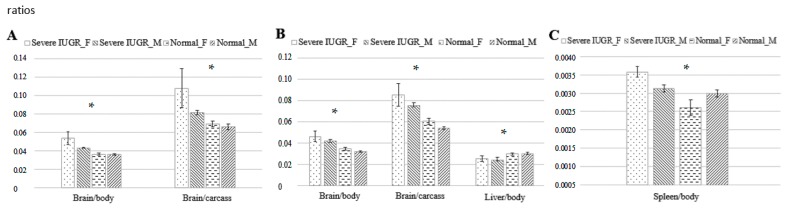
Influence of foetal sex on foetal development under conditions of normal growth or severe IUGR: mean ratios (± mean square error) of brain-to-body weight and brain-to-carcass weight on Gestational Day (GD) 70 (**A**); ratios of brain-to-body weight, brain-to-carcass weight and liver-to-body weight on GD 90 (**B**); and ratio of spleen-to-body weight on GD 90 (**C**). Bars indicate, from left to right, female and male foetuses with severe IUGR, then female and male foetuses showing normal growth. Asterisks indicate the *p* value associated with differences between the foetal sexes and between foetuses showing IUGR or normal growth. * *p* < 0.05.

The comparison, on both GD 70 and 90, of foetuses with severe growth restriction and normal foetuses, showed smaller values than their littermates for body weight (GD 70, 126.5 ± 14.3 g vs. 183.4 ± 4.1 g, *p* < 0.05; GD 90, 404.5 ± 30.9 g vs. 621.8 ± 14.5 g, *p* < 0.005), body length (GD 70, 17.6 ± 0.9 cm vs. 20.5 ± 0.2 cm; GD 90, 27.0 ± 0.9 cm vs. 29.8 ± 0.4 cm; both *p* < 0.05), head weight (GD 70, 35.3 ± 3.2 g vs. 48.2 ± 1 g, *p* < 0.05; GD 90, 103.5 ± 6.3 g vs. 141.9 ± 3.5 g, *p* < 0.005), carcass weight (GD 70, 66.2 ± 8.9 g vs. 100 ± 2.9 g, *p* < 0.05; GD 90, 222.3 ± 18.7 g vs. 364.1 ± 9.6 g, *p* < 0.005) and total weight of viscerae (GD 70, 18.2 ± 2 g vs. 28.3 ± 0.9 g, *p* < 0.01; GD 90, 64.3 ± 7.3 g vs. 98 ± 2.3 g, *p* < 0.05). However, the ratio of brain-to-body weight was significantly higher in foetuses with severe growth restriction than in littermates on GD 70 (0.048 ± 0.004 vs. 0.036 ± 0.001) and GD 90 (0.045 ± 0.004 vs. 0.033 ± 0.001; both *p* < 0.01). This was also observed when only female foetuses were examined (*p* < 0.0005 at both ages; Figure 3). On GD 90, the ratios of brain-to-body weight and brain-to-carcass weight were significantly higher in foetuses with severe growth restriction (both *p* < 0.0005), as were the ratios of liver- and spleen-to-body weight (both *p* < 0.05; Figure 3).

### 2.3. Changes in Foetal Metabolism during Pregnancy and Sex-Related Effects

Table 3 shows measurements of indicators of glucose and lipid metabolism based on markers in plasma, allantoic and amniotic fluids from normal foetuses. Comparison of measurements on GDs 70 and 90 shows that the availability of foetal triglycerides significantly decreased in foetal blood (*p* < 0.05) and allantoic fluid (*p* < 0.005) during pregnancy. Over the same period, the availability of foetal triglycerides in amniotic fluid increased (*p* < 0.05). Low-density lipoprotein cholesterol (LDL-c) in foetal plasma decreased during pregnancy (*p* < 0.005), while HDL-c increased (*p* < 0.001). Total cholesterol decreased in amniotic fluid (*p* < 0.0001) but increased in allantoic fluid (*p* < 0.005). In both these compartments, HDL-c and LDL-c concentrations remained unchanged during pregnancy. Similar results were obtained for the two foetal sexes, except that LDL-c in blood decreased to a greater extent in females than males (*p* < 0.05).

Similar changes to these described in normal foetuses were observed in the subset of foetuses showing severe IUGR, and no sex effects were observed in this case. Foetuses with severe IUGR showed lower plasma LDL-c concentrations than littermates on GD 90 (29.7 ± 2.5 mg/dL vs. 35.8 ± 1.03 mg/dL, *p* < 0.05).

### 2.4. Changes in Foetal Antioxidant/Oxidative Status during Pregnancy and Sex-Related Effects

**Figure 4:**
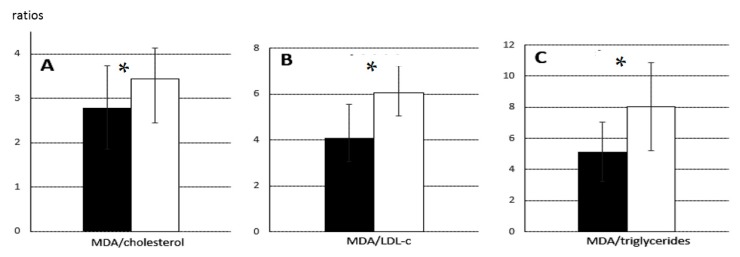
Ratios of MDA to cholesterol, LDL-c and triglycerides of foetuses on Gestational Day (GD) 70 and 90: Mean ratios (± SEM) of: MDA to cholesterol (**A**); MDA to LDL-c (**B**); and MDA to triglycerides (**C**) on GD 70 (black bars) or 90 (white bars). Asterisks indicate significant differences between GD 70 and 90. * *p* < 0.05.

Ferric reducing antioxidant power (FRAP), an index of antioxidant capacity, decreased from GD 70 (10.8 ± 1.3 µmol/mL) to GD 90 (9.9 ± 1.6 µmol/mL, *p* < 0.005) in samples from normal foetuses, yet the concentration of malondialdehyde (MDA) in plasma, an index of total lipid oxidation, decreased during the same period from 20.6 ± 0.4 mmol/mL to 15.8 ± 0.6 mmol/mL (*p* < 0.005). Nevertheless, the ratio of MDA to cholesterol, which takes into account lipid availability, indicated less relative oxidation on GD 70 than GD 90 (2.8 ± 0.9 vs. 3.5 ± 0.7). Similar results were obtained for the ratio of MDA to LDL-c (4.1 ± 1.5 vs. 6.1 ± 1.2) and the ratio of MDA to triglycerides (5.1 ± 1.9 vs. 8.0 ± 2.8) (all *p* < 0.05; Figure 4).

### 2.5. Changes in Foetal Muscle Fatty Acid Composition during Pregnancy and Sex-Related Effects

The neutral and polar fatty acid fractions of the *longissimus dorsi* muscle in normal foetuses varied significantly with gestational age and foetal sex (Table 4 and Table 5). On GD 70, the neutral fraction of female normal foetuses contained significantly more α-linolenic fatty acid (C18:3n-3) and total saturated fatty acids (SFA) than the neutral fraction of male normal foetuses (both *p* < 0.05), as well as less cis-vaccenic fatty acid (C18:1n-7) and lower ratios of ∑n-6/∑n-3 (*p* < 0.01) and monounsaturated fatty acids (MUFA) to SFA (*p* < 0.05). All these sex-related differences disappeared by GD 90.

During pregnancy, content of palmitoleic acid (C16:1n-7) and homo-γ-linolenic acid (C20:3n-6) increased in female normal foetuses (both *p* < 0.001), as did the ratios of ∑n-6/∑n-3 (*p* < 0.01) and C18:1/C18:0 (*p* < 0.005). Similar results were observed for male normal foetuses (∑n-6/∑n-3 *p* < 0.001 and C18:1/C18:0 *p* < 0.01, respectively). The content of several fatty acids decreased significantly during pregnancy, including cis-7 hexadecenoic acid (C16:1n-9), heptadecanoic acid (C17:1) and eicosapentaenoic acid (C20:5n-3) (all *p* < 0.001 in males and *p* < 0.05 in females), as well as stearic acid (C18:0) and erucic acid (C22:1n-9) (all *p* < 0.05 in males and females). 

During pregnancy, male normal foetuses showed increases in myristic acid (C14:0, *p* < 0.05), palmitic acid (C16:0, *p* < 0.001) and linoleic acid (C18:2n-6, *p* < 0.001), while female normal foetuses showed increases in cis-vaccenic acid (C18:1n-7, *p* < 0.01) and total MUFA (*p* < 0.05). During pregnancy, male foetuses showed decreases in mead acid (C20:3n-9, *p* < 0.001), adrenic acid (C22:4n-6, *p* < 0.005), arachidonic acid (C20:4n-6, *p* < 0.01), docosapentaenoic acid (C22:5n-6, *p* < 0.05), total PUFA (*p* < 0.05), and the unsaturated index (UI; *p* < 0.01). Female foetuses showed a significant decrease in α-linolenic acid (*p* < 0.005).

Sex-specific differences were also observed in the composition of the polar fatty acid fraction of normal foetuses. Female foetuses had less palmitoleic, α-linolenic and mead acids than males (all *p* < 0.05) on GD 70 and 90. During pregnancy, several fatty acids increased significantly in both sexes, including stearic, α-linoleic, homo-γ-linolenic and erucic acids (all *p* < 0.0001); docosahexaenoic acid (C22:6n-3, *p* < 0.05); total PUFA (*p* < 0.05); n-6 PUFA (*p* < 0.05); and the C18:1/C18:0 ratio (*p* < 0.0001). Over the same period, several fatty acids decreased significantly in both sexes, including cis-7 hexadecenoic, heptadecanoic, cis-vaccenic, α-linolenic, adrenic and docosapentaenoic acids (all *p* < 0.005); and the MUFA/SFA ratio (*p* < 0.001). Female foetuses showed a significant decrease in heptadecanoic acid (*p* < 0.05), while males showed a significant decrease in mead acid (*p* < 0.05) and an increase in ∑n-6/∑n-3 ratio (*p* < 0.05).

### 2.6. Changes in Foetal Liver Composition during Pregnancy and Sex-Related Effects

Like the intramuscular fatty acid composition, the neutral fatty acid composition of the liver of normal foetuses varied significantly with gestational age and foetal sex (Table 6). During pregnancy, several fatty acids increased in both sexes, including myristic, palmitic, palmitoleic, vaccenic and α-linoleic acids (all *p* < 0.0001); mead acid (*p* < 0.005); adrenic acid (*p* < 0.001); and the C18:1/C18:0 ratio (*p* < 0.005). Over the same period, several fatty acids decreased in both sexes: margaric (C17:0), heptadecanoic, stearic, and α-linolenic acids (all *p* < 0.0001), as well as erucic acid (*p* < 0.0005). In male foetuses, arachidonic and eicosapentaenoic acids decreased (both *p* < 0.05), while in females, total SFA decreased (*p* < 0.01).

On GD 70 and 90, female foetuses showed higher amounts of stearic acid and total SFA (both *p* < 0.05), but lower amounts of cis-7 hexadecenoic acid (*p* < 0.005), oleic acid (*p* < 0.05), and MUFA (*p* < 0.01), as well as lower ratios of C18:1/C18:0 (*p* < 0.01) and MUFA/SFA (*p* < 0.005). On GD 70, females showed higher content of heptadecanoic acid (*p* < 0.05) and lower content of cis-vaccenic acid (*p* < 0.01) than males. On GD 90, females showed lower content of heptadecanoic acid (*p* < 0.05) than males.

In contrast, the composition of the polar fatty acid fraction did not vary significantly between male and female normal foetuses (Table 7), with the exception that α-linoleic acid content was higher in females than males on GD 70 (*p* < 0.05). During pregnancy, both sexes showed increases in arachidonic acid (*p* < 0.0005); margaric, stearic, linoleic, and mead acids (all *p* < 0.0001); myristic acid (*p* < 0.05); total PUFA and n-6 PUFA (both *p* < 0.0001); and UI (*p* < 0.005). During the same period, both sexes showed decreases in α-linolenic, erucic, cis-7 hexadecenoic and heptadecanoic acids (all *p* < 0.0001); oleic acid (*p* < 0.005); vaccenic acid (*p* < 0.05); total MUFA (*p* < 0.0001); and the ratios C18:1/C18:0 and MUFA/SFA (both *p* < 0.0005).

## 3. Discussion

The results of the present study support the prominent role of the maternal metabolic profile on foetal development and homeorhesis and provide new insights on the effects of offspring sex on fetoplacental development, lipid availability and fatty acid composition at non-adipose tissues involved in metabolism regulation (muscle and liver). These data may be considered relevant for humans since despite differences in placentation, human and pig foetuses and pregnant females show similar lipid metabolism and distribution [39].

In the current study, lower cholesterol concentration in maternal plasma correlated with deficiencies in foetal development, which it is consistently with previous studies on GD 42 [16]. In contrast, poor availability of glucose, the main energy source for developing foetuses [40], affected foetal development on GD 90 but not on GD 70 in our study, consistent with reports that hypoglycaemia in early development is non-critical and can be compensated [41].

The higher growth of the brain when compared to total body observed in the foetuses affected by IUGR is consistent with the “brain-sparing effect” firstly described by Rudolph (1984) [42]. There was also a higher growth of other organs (mainly heart, liver and spleen), which is also consistent with studies in humans documenting “heart- and liver-sparing” cardiovascular adaptations—analogous to the brain-sparing effect—in response to foetal malnutrition and hypoxia [43,44]. Previous studies address that changes in the growth, morphology and function of various organs are dependant on the timing and severity of nutritional restriction [45,46], with specifically a determinant effect triggered by lipid availability. At the same time, our results support previous work indicating sex-specific differences in the growth of the different organs [27,31].

Maternal undernutrition in our study clearly affected foetal lipid availability. The developing foetus and placenta require large amounts of lipids for: (a) synthesis of cholesterol, which acts as a key constituent of cell membranes and organelles and is the precursor of a range of hormones and metabolic regulators necessary for successful pregnancy [47,48,49,50]; (b) secretion of key products such as lipoproteins; and (c) storage of triglycerides [47,48,49]. Although placental and foetal tissues can synthesize lipids de novo [47,51], the building blocks must be taken up by the placenta, i.e. maternal free fatty acids; triglycerides, which placental lipases hydrolyse into fatty acid constituents; and lipoprotein-associated cholesterol [19,21,23,51]. Triglycerides are a major source of energy for the foetus [52,53], but a balance is needed: low levels of triglycerides delay growth, while high levels can cause foetal macrosomia [54]. Availability of lipids to the fetoplacental unit depends on de novo synthesis by the foetus as well as maternal transfer. Cholesterol, for example, can reach the foetal circulatory system after crossing the syncytiotrophoblast as LDL-c [49,55]. In pig foetuses, fatty acid availability and composition depend more on foetal synthesis from precursors transferred from the mother than on direct maternal transfer [56,57,58,59,60,61], since fatty acids do not easily cross the placenta in ruminants, pigs or horses [62].

Lipid availability to the foetoplacental unit also depends on the antioxidant/oxidative status of the foetus. Our results suggest that, during pregnancy, antioxidant capacity decreases and lipid peroxidation increases in foetuses affected by maternal undernutrition, as suggested by previous studies [24,25,26]. These changes may explain the lower lipid availability later in pregnancy.

Cholesterol and triglyceride availability in our study did not differ significantly between male and female foetuses, consistently with prior studies [56]. Our data further show that the two sexes showed similar antioxidant capacity and lipid peroxidation. In other words, foetal development was affected to a much greater extent by gestational age—and therefore by nutritional restriction—than by sex. Nevertheless, we did observe sex-related differences in how the fatty acid composition of non-adipose tissues involved in metabolism regulation (muscle and liver) changed between GD 70 and 90 in foetuses affected by maternal undernutrition.

Our data indicate significant sex-related differences in content of essential fatty acids, which are so-called because they must be obtained from maternal transfer in the case of foetuses [22,63] or from diet in the case of adults [64]. The major essential fatty acids are linolenic PUFA (an omega-3 fatty acid) and linoleic PUFA (an omega-6 fatty acid); the long-chain omega-3 PUFA eicosapentaenoic and docosahexaenoic acids and the long-chain omega-6 PUFA gamma-linolenic and arachidonic acids are also essential. Future work should examine the maternal and/or placental factors that may drive the sex-dependent differences in essential fatty acid availability that we observed.

Most of the differences in fatty acid composition between male and female foetuses that we observed in muscle and liver belonged to the neutral fraction corresponding to triglycerides, which are an essential energy source [19]. Smaller differences were observed in the polar fraction corresponding to phospholipids, which constitute cell membranes and are essential for tissue development [19], so sex-related differences in fatty acid composition are most related to energy partitioning than to organ development.

We found that male foetuses had a higher n-6/n-3 ratio than females, and a high n-6/n-3 ratio appears to be deleterious [65], corresponding to the prodromal phase of insulin resistance [66]. Optimal development depends on adequate availability of n-6 [67], while n-3 may improve insulin function. Our results are consistent with previous reports that alterations in lipid metabolism and insulin regulation appear early in the development of male foetuses under limited nutrition [68,69,70,71]. Indeed, males in our study showed a significantly higher ratio of MUFA to SFA in muscle triglycerides than females on GD 70, although this difference was no longer significant on GD 90. On GD 70 and 90, males showed lower stearic acid content in the liver, and this acid is considered to protect metabolic health [72,73]. Males also showed higher ratios of MUFA to SFA and C18:1 to C18:0, indicating higher stearoyl-CoA desaturase activity. Although we observed few sex-related differences in the changes in phospholipid composition of muscle or liver during pregnancy, we did observe that females had higher content of linoleic acid in the liver than males on GD 70, and that males had higher content of mead acid in the muscle on GD 70 and 90. The greater content of mead acid in males likely reflects a worse homeorhesis state, since synthesis of mead or eicosatrienoic acid occurs in response to severe deficiency of fatty acids, mainly linoleic acid [74,75].

## 4. Material and Methods

### 4.1. Ethics Statement

These experiments were performed according to the Spanish Policy for Animal Protection RD53/2013, which complies with the European Union Directive 2010/63/UE on the protection of animals used for research. The experimental procedures were specifically assessed and approved by the INIA Committee of Ethics in Animal Research (report CEEA 2013/036, finally approved on 19 February 2014). Sows were housed in INIA animal facilities, which meet local, national and European requirements for Scientific Procedure Establishments.

### 4.2. Animals and Experimental Procedures

The study involved a total of 56 foetuses obtained from 9 multiparous purebred Iberian sows with an average body weight of 147.7 ± 16.0 kg, which became pregnant after cycle synchronization with altrenogest (Regumate^®^, MSD, Boxmeer, The Netherlands) and insemination with cooled semen from a purebred Iberian boar. All sows and the boar were homozygous for the *LEPRc.1987T* allele based on pyrosequencing [76].

Sows were fed with a standard grain-based food diet with the following mean component values: dry matter, 89.8%; crude protein, 15.1%; fat, 2.8%; and metabolizable energy, 3.0 Mcal/kg. Diet analysis (Table 8) showed that the most abundant fatty acids (FA) were palmitic (18.7%), oleic (23.2%) and linoleic acids (46.5%).

The amount of food was adjusted to fulfil individual daily maintenance requirements, based on data from the British Society of Animal Science, from the start of the experimental period until GD 35 [77]. On this day, all sows were weighed and the amount of food offered to each sow was adjusted to fulfil 50% of their daily maintenance requirements, which has been shown to increase IUGR incidence [78]. Every day, a single food ration was weighed out and given to each sow in her individual pen; hence, the diet of each female was adjusted to her own weight.

Foetuses were obtained on GD 70 from five sows or on GD 90 from four sows. These gestational time points correspond to approximately 60% and 80% of a 112-day gestation typical for this breed, corresponding to 24 and 32 weeks of human pregnancy, respectively. These time points were chosen because until GD 70 of swine pregnancy, lipid anabolism and foetal metabolism resemble maternal metabolism [79]; around GD 90, foetal metabolism is independently regulated, and foetal development is more affected by nutrient availability [80].

On GD 70 and 90, blood samples were drawn from the orbital sinus of sows that had fasted for approximately 16 h. Samples were collected in sterile, heparinised 4-mL vacuum tubes (Vacutainer™ Systems Europe, Meylan, France) and were immediately centrifuged at 1500 *g* for 15 min. The plasma was separated and biobanked into polypropylene vials at −80 °C until they were assayed for metabolic biomarkers including glycaemic values and lipid profile.

### 4.3. Sampling of Placentas and Foetuses

Animals were euthanised by stunning and exsanguination, in compliance with RD53/2013 standard procedures, and the entire genital tracts were immediately collected for morphometric evaluation and foetal sampling. The contents of the uterus were exposed, and conceptus position was recorded. In each normal non-IUGR foetus, the allantoic and amniotic fluids were obtained by aspiration through the chorioallantoic and amniochorionic membranes, and blood samples were drawn from the heart and/or umbilical cord using heparinised syringes. Blood was processed as described above for sows, while allantoic and amniotic fluids were centrifuged at 1500× *g* for 15 min and supernatants were biobanked into polypropylene vials at −80 °C until they were assayed for metabolic biomarkers (including glycaemic values and lipid profile) and antioxidant/oxidative status. Rectangular sections of the uterine wall were collected, fixed in 4% paraformaldehyde, embedded in paraffin and stained using hematoxylin-eosin. Individual sections of the placentas were measured morphometrically as described [81] to obtain the average width of the placental folds, which served as an index of placental maturation.

### 4.4. Evaluation of Foetal Sex and Morphological Features

Foetal sex was determined by visual inspection immediately after recovery; detailed pregnancy-by-pregnancy information is shown in Table 9. Body length (crown-rump length), head size (occipito-nasal length and biparietal diameter) and corpulence (thoracic and abdominal circumferences) were measured in all the normal and IUGR foetuses. Total foetus weight was determined, then the head was separated from the trunk at the atlanto-occipital union, and head and trunk were weighed separately. All viscerae were obtained and weighed together immediately, and then the brain, heart, lungs, liver, intestine, kidneys, spleen and pancreas were weighed separately. Ratios of head-to-body weight, brain-to-head weight, and the weight of total viscera and individual organs (brain, heart, lungs, liver, kidneys, intestine, pancreas, and spleen) relative to body weight were calculated.

Samples from liver, kidneys, duodenum and ileum were fixed in 4% paraformaldehyde, embedded in paraffin and stained with hematoxylin-eosin. The degree of maturation of these organs was assessed by evaluating the number of glomeruli with a well-defined glomerular tuft in the kidney [82], the decrease of hepatic haematopoiesis in the liver [83] and villus height and crypt depth in the intestine [84]. This assessment was made by an investigator blinded to sow and foetus details.

### 4.5. Evaluation of Maternal and Foetal Metabolic Status

Lipid profile parameters (triglycerides, total cholesterol, HDL-c, LDL-c) were measured in maternal plasma as well as in plasma, allantoic and amniotic fluids of normal foetuses. Assays were performed using a clinical chemistry analyser (Saturno 300 plus, Crony Instruments s.r.l., Rome, Italy), according to the manufacturer’s instructions.

### 4.6. Evaluation of Foetal Adiposity and Fat Composition

Total fat was quantified in carcasses, and the total fat percentage and fatty acid composition (in g/100) were determined in intramuscular fat and liver of normal foetuses. For this purpose, samples from *longissimus dorsi* muscle and the left liver lobe were biobanked at −80 °C until they were assayed as described [85]. Intramuscular fat and liver fat were extracted from 300 mg of lyophilised and homogenised samples using the Ball-mill procedure [86]. Fatty acids in the total lipid extracts were identified and quantified by gas chromatography (HP6890, Hewlett Packard, Avondale, PA, USA) after methylation, as described in [87,88]. Fatty acid methyl esters were fractionated on a cross-linked polyethylene glycol capillary column (30 cm × 0.32 mm × 0.25 μm, Hewlett Packard Innowax) and a temperature gradient from 170 °C to 245 °C. The injector and detector were maintained at 250 °C. Neutral lipid fractions (triglycerides) and polar lipid fractions (phospholipids) were analysed using gas chromatography after passing them through aminopropyl minicolumns previously activated with 7.5 mL of hexane as described [89]. The percentages of individual fatty acids were used to calculate proportions of SFA, MUFA and PUFA, as well as total n-3 and n-6 and their ratio (∑n-6/∑n-3). The unsaturation index (UI) was obtained from the ratio of MUFA to SFA, and the activity of the stearoyl-CoA desaturase enzyme 1 was inferred from the ratio of the enzyme product, oleic acid (C18:1n-9), to the enzyme substrate, stearic acid (C18:0).

### 4.7. Evaluation of Foetal Antioxidant/Lipid Oxidative Status

In normal foetuses, values for total antioxidant capacity were assayed using the ferric reducing antioxidant power assay (FRAP) as previously described [90], while lipid peroxidation was assessed by measuring MDA (mmol/mL) using the thiobarbituric acid reaction and HPLC separation with fluorescence detection as previously described [91].

### 4.8. Statistical Analyses

Data were analysed using SPSS 22.0 (IBM Corp., Armonk, NY, USA). Based on previous studies [42], foetuses with severe growth restriction were defined as those with a body weight more than one standard deviation below the litter mean value. Among the 33 foetuses collected on GD 70, four (11%) were classified as showing severe growth restriction. Among the 23 foetuses collected on GD 90, four (17.4%) were so classified. Effects of gestational age (GD 70 vs. 90), sex (female vs. male) and growth restriction on developmental traits, adiposity, fatty-acid composition, and metabolic and foetal oxidative status were assessed by two-way ANOVA. Duncan’s post-hoc test was performed to check differences among groups in multiple comparisons. Relationships between maternal metabolic biomarkers and features of foetuses showing normal growth were explored using Pearson correlation. The sow was considered the experimental unit for all variables in order to avoid biasing the results according to litter size: foetuses with the same sex and development (normal or IUGR) from the same sow were averaged together, giving one data point per sow. All results were expressed as mean ± SEM and statistical significance was accepted from *p* < 0.05.

## 5. Conclusions

The present study supports the importance of the maternal lipid profile and placental transfer of lipids, mainly essential fatty acids, for foetal development, and it confirms and extends previous studies suggesting that female foetuses show better adaptive responses in the form of greater synthesis of non-essential fatty acids and better transfer of essential fatty acids, resulting in better metabolic indexes. These sex-related differences were observed primarily in the neutral lipid fraction (triglycerides), suggesting a strong influence of sex on postnatal energy partitioning. These results may help guide future studies on understanding and optimising the maternal diet and placenta transfer capacity in order to meet foetal requirements for fatty acids and prevent the postnatal problems associated with insufficient prenatal fatty acid availability.

## Figures and Tables

**Table 1:** Effect of age and sex on absolute weights and body measurements of male and female foetuses at Gestational Day (GD) 70 and 90. Mean values for morphometric measurements and absolute body and main organs weights at GD 70 and 90 in female (F) and male (M) foetuses.

		70		90		MSE ^a^	*p*-Value				
							Same Age F vs. M		Same Sex 70 vs. 90		Age × Sex
		F	M	F	M		70	90	F	M	
**Body morphometry**	**Body length (cm)**	20.2	20.2	29.1	29.5	0.495	0.343	0.746	<0.001	<0.001	<0.001
	**Occipito-nasal length (cm)**	7.7	6.6	8.4	9.2	0.403	0.045	0.193	0.241	<0.001	<0.001
	**Biparietal diameter (cm)**	3.0	2.9	3.9	4.1	0.071	0.748	0.173	<0.001	<0.001	<0.001
	**Trunk diameter (cm)**	2.7	2.9	4.2	4.6	0.117	0.177	0.028	<0.001	<0.001	<0.001
	**Trunk circumference (cm)**	11.0	11.4	16.7	17.5	0.355	0.222	0.221	<0.001	<0.001	<0.001
	**Abdominal circumference (cm)**	9.3	9.8	14.5	15.3	0.439	0.294	0.277	<0.001	<0.001	<0.001
**Body weight**	**Body weight (g)**	171.9	180.1	542.0	616.4	18.969	0.453	0.09	<0.001	<0.001	<0.001
	**Head weight (g)**	46.8	46.6	130.4	138.9	4.086	0.948	0.340	<0.001	<0.001	<0.001
	**Carcass weight (g)**	91.3	99.1	309.2	362.7	12.160	0.268	0.06	<0.001	<0.001	<0.001
**Viscerae and main organs weight**	**Brain weight (g)**	6.5	6.5	19.9	19.9	0.378	0.968	0.996	<0.001	<0.001	<0.001
	**Viscerae weight (g)**	26.3	27.7	86.5	96.5	3.245	0.495	0.168	<0.001	<0.001	<0.001
	**Heart weight (g)**	1.4	1.5	4.7	5.2	0.193	0.616	0.233	<0.001	<0.001	<0.001
	**Lungs weight (g)**	6.1	6.3	17.4	20.4	0.691	0.662	0.072	<0.001	<0.001	<0.001
	**Liver weight (g)**	7.8	7.7	15.8	18.4	0.905	0.827	0.172	<0.001	<0.001	<0.001
	**Intestine weight (g)**	5.2	4.9	21.7	25.2	1.089	0.599	0.187	<0.001	<0.001	<0.001
	**Spleen weight (g)**	0.2	0.2	1.2	1.5	0.053	0.425	0.018	<0.001	<0.001	<0.001
	**Kidneys weight (g)**	2.2	2.2	5.6	6.1	0.260	0.951	0.375	<0.001	<0.001	<0.001

^a^ MSE = Mean square error.

**Table 2:** Effects of gestational time and sex on foetal adiposity. Mean values (%) for fat content in the *longissimus dorsi* muscle (LD) and liver at Gestational Day (GD) 70 and 90 in female (F) and male (M) foetuses.

	70		90		MSE ^a^	*p*-Value			
						Same Age F vs. M		Same Sex 70 vs. 90	
	F	M	F	M		70	90	F	M
**Fat in LD (%)**	0.09	0.08	0.07	0.08	0.003	0.605	0.681	0.293	0.641
**Fat in Liver (%)**	0.14	0.14	0.14	0.14	0.002	0.434	0.522	0.814	0.294
**Fat in carcass**	0.08	0.09	0.08	0.09	0.002	0.107	0.070	0.069	0.660

^a^ MSE = Mean square error.

**Table 3:** Effect of age and sex on foetal metabolism. Mean values for parameters of lipids and glucose metabolism at Gestational Day (GD) 70 and 90 in female (F) and male (M) foetuses.

			70		90		MSE ^a^	*p*-Value				
			F	M	F	M		Same Age F vs. M		Same Sex 70 vs. 90		Age × Sex
								70	90	F	M	
**Glucose metabolism**	**Glucose (mg/dL)**	**Foetal plasma**	24.3	19.8	64.2	58.6	5.742	0.480	0.748	0.011	<0.001	<0.001
		**Amniotic fluid**	29.6	23.1	24.3	26.1	1.038	0.017	0.598	0.063	0.310	0.112
		**Allantoic fluid**	7.5	5.3	21.9	22.0	1.352	0.233	0.976	<0.001	<0.001	<0.001
	**Fructosamine (mg/dL)**	**Foetal plasma**	134.8	129.6	107.8	114.9	3.147	0.583	0.190	0.003	0.094	0.012
		**Amniotic fluid**	48.0	44.2	57.3	57.7	1.216	0.157	0.879	0.002	<0.001	<0.001
		**Allantoic fluid**	90.2	81.6	270.7	340.8	27.390	0.592	0.568	0.016	0.001	<0.001
**Lipid metabolism**	**Triglycerides (mg/dL)**	**Foetal plasma**	39.4	32.2	28.3	28.6	1.804	0.204	0.938	0.081	0.437	0.154
		**Amniotic fluid**	1.8	2.5	3.6	6.3	0.701	0.070	0.435	0.048	0.114	0.113
		**Allantoic fluid**	10.6	11.3	4.9	5.8	0.857	0.793	0.509	0.024	0.024	0.012
	**Total cholesterol (mg/dL)**	**Foetal plasma**	65.1	58.7	63.2	59.6	1.636	0.160	0.438	0.743	0.823	0.489
		**Amniotic fluid**	4.0	3.9	2.8	2.2	0.180	0.885	0.082	0.007	0.001	<0.001
		**Allantoic fluid**	2.7	2.5	4.5	6.3	0.417	0.636	0.310	0.051	0.004	0.003
	**HDL-c (mg/dL)**	**Foetal plasma**	12.8	13.8	21.2	18.7	0.624	0.482	0.072	<0.001	<0.001	<0.001
		**Amniotic fluid**	2.2	2.5	2.1	2.4	0.068	0.189	0.116	0.529	0.507	0.191
		**Allantoic fluid**	2.1	2.4	2.2	2.2	0.085	0.145	0.971	0.798	0.272	0.447
	**LDL-c (mg/dL)**	**Foetal plasma**	45.4	41.0	33.3	35.7	1.261	0.225	0.252	0.005	0.07	0.004
		**Amniotic fluid**	3.3	3.5	3.6	3.5	0.094	0.415	0.664	0.302	0.864	0.783
		**Allantoic fluid**	3.1	3.4	3.2	4.3	0.233	0.257	348	0.833	0.256	0.349

^a^ MSE = Square Mean error.

**Table 4:** Effects of age and sex on foetal neutral fatty-acid composition of *longissimus dorsi* muscle. Mean values for neutral lipids in the *longissimus dorsi* muscle at Gestational Day (GD) 70 and 90 in female (F) and male (M) foetuses.

	70		90		MSE ^a^	*p*-Value				
						Same Age F vs. M		Same Sex 70 vs. 90		Age × Sex
Fatty Acids (g/100 g Total Fatty Acids)	F	M	F	M		70	90	F	M	
**C14:0**	3.59	3.42	3.75	3.74	0.064	0.383	0.632	0.506	0.036	0.181
**C16:0**	28.97	26.79	29.33	29.25	0.375	0.069	0.549	0.809	0.001	0.021
**C16:1n-9**	4.22	4.35	3.46	3.38	0.102	0.625	0.46	0.018	<0.001	<0.001
**C16:1n-7**	3.78	4.3	7.33	7.28	0.251	0.198	0.563	<0.001	<0.001	<0.001
**C17:0**	0.35	0.45	0.27	0.28	0.031	0.245	0.73	0.333	0.072	0.107
**C17:1**	2.96	2.82	1.68	1.77	0.089	0.364	0.177	<0.001	<0.001	<0.001
**C18:0**	13.55	13	12.21	11.55	0.218	0.351	0.375	0.035	0.012	0.006
**C18:1n-9**	21.51	21.87	22.09	22.55	0.222	0.583	0.585	0.507	0.168	0.453
**C18:1n-7**	6.36	6.86	7.34	7.17	0.088	0.029	0.234	0.001	0.109	0.001
**C18:2n-6**	3.34	3.19	3.64	3.96	0.079	0.363	0.163	0.151	<0.001	0.001
**C18:3n-3**	0.26	0.17	0.11	0.15	0.015	0.016	0.386	0.004	0.478	0.003
**C20:3n-9**	0.57	0.63	0.56	0.54	0.010	0.063	0.147	0.757	<0.001	0.003
**C20:3n-6**	0.24	0.26	0.43	0.47	0.016	0.569	0.098	<0.001	<0.001	<0.001
**C20:4n-6**	5.23	6.49	4.09	4.19	0.317	0.19	0.499	0.223	0.007	0.012
**C20:5n-3**	0.29	0.28	0.2	0.19	0.008	0.718	0.605	<0.001	<0.001	<0.001
**C22:1n-9**	0.32	0.3	0.21	0.2	0.015	0.731	0.796	0.024	0.01	0.007
**C22:4n-6**	1.44	1.68	1.05	1.11	0.078	0.297	0.219	0.125	0.003	0.006
**C22:5n-6**	0.91	1.11	0.89	0.91	0.037	0.079	0.567	0.884	0.022	0.067
**C22:5n-3**	0.37	0.35	0.3	0.3	0.017	0.701	0.621	0.171	0.276	0.373
**C22:6n-3**	1.46	1.67	1.06	1.03	0.221	0.366	0.862	0.227	0.001	0.147
**SFA ^1^**	46.45	43.66	45.55	44.81	0.407	0.029	0.345	0.566	0.166	0.063
**MUFA ^2^**	39.14	40.51	42.12	42.35	0.394	0.231	0.842	0.027	0.054	0.016
**PUFA ^3^**	15.01	15.83	12.34	12.84	0.492	0.568	0.307	0.085	0.021	0.027
**UI ^4^**	0.98	1.01	0.87	0.88	0.021	0.546	0.37	0.123	0.008	0.021
**PUFAn-3 ^5^**	3.27	2.48	1.67	1.67	0.232	0.3	0.686	0.143	0.001	0.057
**PUFAn-6 ^6^**	2.59	3.05	2.37	2.49	0.108	0.173	0.249	0.558	0.037	0.084
**∑n-6/∑n-3**	0.98	1.24	1.45	1.54	0.044	0.006	0.517	0.003	<0.001	<0.001
**C18:1/C18:0**	2.08	2.24	2.43	2.62	0.05	0.158	0.388	0.009	0.009	0.001
**MUFA/SFA**	0.85	0.93	0.93	0.95	0.013	0.046	0.618	0.135	0.488	0.052

^a^ MSE = Mean square error. ^1^ SFA = Saturated fatty acids; Includes: C14:0, C16:0, C17:0 and C18:0. ^2^ MUFA = Monounsaturated fatty acids; Includes: C16:1n-9, C16:1n-7, C17:1, C18:1n-9, C18:1n-7 and C22:1n-9. ^3^ PUFA = Polyunsaturated fatty acids: Includes: C18:2n-6, C18:3n-3, C20:3n-9, C20:3n-6, C20:4n-6, C20:5n-3, C22:4n-6, C22:5n-6, C22:5n-3 and C22:6n-3. ^4^ UI = Conversion index. ^5^ Includes: C18.2n-6, C20:4n-6 and C22:4n-6. ^6^ Includes: C18:3n-3, C20:5n-3 and C22:6n-3.

**Table 5:** Effects of age and sex on foetal polar fatty-acid composition of *longissimus dorsi* muscle. Mean values for polar lipids in the *longissimus dorsi* muscle at Gestational Day (GD) 70 and 90 in female (F) and male (M) foetuses.

	70		90		MSE ^a^	*p*-Value				
						Same Age F vs. M		Same Sex 70 vs. 90		Age × Sex
Fatty Acids (g/100 g Total Fatty Acids)	F	M	F	M		70	90	F	M	
**C14:0**	1.99	1.83	1.83	1.84	0.067	0.396	0.944	0.448	0.95	0.827
**C16:0**	23.69	23.68	23.79	23.15	0.103	0.967	0.082	0.747	0.062	0.113
**C16:1n-9**	2.75	2.69	2.05	1.95	0.077	0.724	0.618	0.001	<0.001	<0.001
**C16:1n-7**	2.92	3.2	3.12	3.03	0.058	0.053	0.645	0.193	0.327	0.308
**C17:0**	1.11	1.06	0.99	1.06	0.031	0.659	0.42	0.018	0.977	0.713
**C17:1**	2.34	2.34	1.45	1.55	0.061	0.944	0.088	<0.001	<0.001	<0.001
**C18:0**	11.15	11.04	11.77	11.82	0.083	0.591	0.801	0.013	<0.001	<0.001
**C18:1n-9**	21.16	21.46	21.63	21.77	0.127	0.433	0.989	0.14	0.413	0.415
**C18:1n-7**	6.7	6.63	6.28	6.13	0.058	0.606	0.273	0.001	0.002	<0.001
**C18:2n-6**	2.76	2.61	4.03	4.19	0.110	0.235	0.44	<0.001	<0.001	<0.001
**C18:3n-3**	0.14	0.17	0.12	0.13	0.007	0.127	0.512	0.102	0.065	0.036
**C20:1n-9**	0.7	0.76	0.84	0.85	0.016	0.229	0.804	0.004	0.024	0.003
**C20:3n-9**	0.58	0.63	0.63	0.56	0.010	0.015	0.016	0.068	0.007	0.012
**C20:3n-6**	0.23	0.25	0.57	0.67	0.030	0.382	0.14	<0.001	<0.001	<0.001
**C20:4n-6**	12.86	12.76	12.76	13.21	0.092	0.595	0.103	0.716	0.075	0.252
**C20:5n-3**	0.31	0.31	0.3	0.27	0.008	0.974	0.252	0.757	0.055	0.181
**C22:1n-9**	0.55	0.56	0.78	0.74	0.018	0.847	0.176	<0.001	<0.001	<0.001
**C22:4n-6**	3.02	2.97	2.01	2.11	0.070	0.541	0.074	<0.001	<0.001	<0.001
**C22:5n-6**	1.35	1.38	1.19	1.24	0.024	0.704	0.65	0.02	0.038	0.019
**C22:5n-3**	0.72	0.68	0.69	0.62	0.015	0.308	0.285	0.526	0.157	0.180
**C22:6n-3**	2.98	2.99	3.16	3.09	0.028	0.932	0.355	0.055	0.148	0.101
**SFA ^1^**	37.94	37.63	38.39	37.87	0.692	0.447	0.261	0.216	0.568	0.467
**MUFA ^2^**	37.12	37.63	36.15	36.03	0.681	0.256	0.427	0.002	0.002	0.904
**PUFA ^3^**	24.95	24.74	25.47	26.09	0.477	0.401	0.215	0.219	0.002	0.195
**UI ^4^**	1.39	1.39	1.37	1.39	0.025	0.879	0.257	0.24	0.815	0.655
**PUFAn-3 ^5^**	4.15	4.15	4.27	4.11	0.083	0.993	0.268	0.329	0.725	0.568
**PUFAn-6 ^6^**	20.23	19.96	20.56	21.42	0.399	0.283	0.118	0.448	0.001	0.123
**∑n-6/∑n-3**	4.9	4.84	4.84	5.24	0.064	0.647	0.081	0.769	0.016	0.085
**C18:1/C18:0**	0.45	0.43	0.48	0.47	0.004	0.168	0.908	0.001	0.001	<0.001
**MUFA/SFA**	0.98	1	0.94	0.95	0.008	0.298	0.733	0.008	0.031	0.013

^a^ MSE = Mean square error. ^1^ SFA = Saturated fatty acids; Includes: C14:0, C16:0, C17:0 and C18:0. ^2^ MUFA = Monounsaturated fatty acids; Includes: C16:1n-9, C16:1n-7, C17:1. C18:1n-9, C18:1n-7, C20:1n-9 and C22:1n-9. ^3^ PUFA = Polyunsaturated fatty acids: Includes: C18:2n-6, C18:3n-3, C20:3n-9, C20:3n-6, C20:4n-6, C20:5n-3, C22:4n-6, C22:5n-6, C22:5n-3 and C22:6n-3. ^4^ UI = Conversion index. ^5^ Includes: C18.2n-6, C20:4n-6 and C22:4n-6. ^6^ Includes: C18:3n-3, C20:5n-3 and C22:6n-3.

**Table 6:** Effects of age and sex on foetal neutral fatty-acid composition of liver. Mean values for neutral lipids in the liver at Gestational Day (GD) 70 and 90 in female (F) and male (M) foetuses.

	70		90		SEM ^a^	*p*-Value				
						Same Age F vs. M		Same Sex 70 vs. 90		Age × Sex
Fatty Acids (g/100 g Total Fatty Acids)	F	M	F	M		70	90	F	M	
**C14:0**	1.82	1.61	2.93	3.24	0.128	0.391	0.378	<0.001	<0.001	<0.001
**C16:0**	19.02	19.67	23.14	23.25	0.336	0.345	0.945	<0.001	<0.001	<0.001
**C16:1n-9**	1.58	2.03	1.84	2.08	0.063	0.016	0.153	0.101	0.77	0.016
**C16:1n-7**	6.8	7.31	8.66	9.34	0.196	0.192	0.289	<0.001	<0.001	<0.001
**C17:0**	3.5	3.06	1.6	1.59	0.125	0.028	0.887	<0.001	<0.001	<0.001
**C17:1**	2.19	2.29	1.22	1.36	0.072	0.392	0.049	<0.001	<0.001	<0.001
**C18:0**	22.89	18.94	16.22	14.77	0.656	0.031	0.191	0.001	0.008	<0.001
**C18:1n-9**	18.94	19.8	17.67	20.68	0.376	0.365	0.04	0.249	0.383	0.059
**C18:1n-7**	5.6	6.42	7.16	6.87	0.125	0.011	0.26	<0.001	0.166	<0.001
**C18:2n-6**	2.29	2.39	3.24	3.14	0.085	0.533	0.92	<0.001	<0.001	<0.001
**C18:3n-3**	0.37	0.36	0.26	0.26	0.010	0.783	0.808	<0.001	<0.001	<0.001
**C20:1n-9**	0.26	0.41	0.24	0.25	0.045	0.361	0.998	0.6	0.319	0.451
**C20:3n-9**	0.29	0.29	0.37	0.32	0.009	0.841	0.216	0.003	0.126	0.004
**C20:4n-6**	12.16	12.99	12.98	10.61	0.373	0.344	0.169	0.488	0.018	0.084
**C20:5n-3**	0.1	0.11	0.08	0.26	0.033	0.811	0.3	0.411	0.172	0.221
**C22:1n-9**	0.47	0.37	0.28	0.24	0.022	0.087	0.689	0.007	0.011	0.001
**C22:4n-6**	0.43	0.43	0.66	0.54	0.023	0.938	0.169	0.011	0.012	0.001
**C22:5n-3**	0.84	1.12	0.95	0.78	0.049	0.069	0.17	0.334	0.025	0.038
**C22:6n-3**	0.43	0.4	0.51	0.4	0.018	0.519	0.194	0.152	0.918	0.164
**SFA ^1^**	47.23	43.28	43.89	42.85	0.467	0.009	0.029	0.009	0.726	0.002
**MUFA ^2^**	35.84	38.63	37.07	40.83	0.564	0.04	0.107	0.445	0.138	0.014
**PUFA ^3^**	16.93	18.09	19.04	16.32	0.469	0.314	0.215	0.157	0.164	0.234
**UI ^4^**	1	1.08	1.08	1.01	0.016	0.087	0.253	0.093	0.128	0.145
**SUMN3 ^5^**	1.75	1.99	1.8	1.7	0.063	0.155	0.793	0.697	0.138	0.267
**SUMN6 ^6^**	14.89	15.81	16.88	14.3	0.422	0.362	0.204	0.151	0.179	0.220
**N6/N3**	8.58	8.23	9.41	8.77	0.199	0.463	0.453	0.108	0.357	0.225
**SUMC18:1/C18:0**	1.12	1.47	1.57	1.93	0.063	0.011	0.103	0.003	0.006	<0.001
**MUFA/SFA**	0.77	0.91	0.85	0.95	0.019	0.007	0.067	0.096	0.321	0.002

^a^ MSE = Mean square error. ^1^ SFA = Saturated fatty acids; Includes: C14:0, C16:0, C17:0 and C18:0. ^2^ MUFA = Monounsaturated fatty acids; Includes: C16:1n-9, C16:1n-7, C17:1. C18:1n-9, C18:1n-7 and C22:1n-9. ^3^ PUFA = Polyunsaturated fatty acids: Includes: C18:2n-6, C18:3n-3, C20:3n-9, C20:3n-6, C20:4n-6, C20:5n-3, C22:4n-6 C22:5n-6, C22:5n-3 and C22:6n-3. ^4^ UI = Conversion index. ^5^ Includes: C18.2n-6, C20:4n-6 and C22:4n-6. ^6^ Includes: C18:3n-3, C20:5n-3 and C22:6n-3.

**Table 7:** Effects of age and sex on foetal polar fatty-acid composition of liver. Mean values (%) for polar lipids in the liver at Gestational Day (GD) 70 and 90 in female (F) and male (M) foetuses.

	70		90		MSE ^a^	*p*-Value				
						Same Age F vs. M		Same Sex 70 vs. 90		Age × Sex
Fatty Acids (g/100 g Total Fatty Acids)	F	M	F	M		70	90	F	M	
**C14:0**	1.73	1.66	1.89	1.8	0.033	0.45	0.524	0.168	0.084	0.088
**C16:0**	22.83	21.93	23.13	22.39	0.217	0.184	0.188	0.689	0.389	0.215
**C16:1n-9**	1.77	1.73	1.34	1.39	0.038	0.638	0.197	0.001	<0.001	<0.001
**C16:1n-7**	5.11	4.84	5.11	4.96	0.064	0.162	0.561	0.999	0.502	0.351
**C17:0**	3.09	3.05	1.72	1.75	0.100	0.764	0.797	<0.001	<0.001	<0.001
**C17:1**	1.7	1.7	0.86	0.83	0.064	0.988	0.435	<0.001	<0.001	<0.001
**C18:0**	18	18.08	19.17	19.93	0.165	0.819	0.165	0.014	<0.001	<0.001
**C18:1n-9**	14.09	13.7	12.08	12.18	0.274	0.583	0.963	0.021	0.032	0.016
**C18:1n-7**	8.03	8.19	7.28	7.13	0.204	0.784	0.727	0.176	0.077	0.158
**C18:2n-6**	2.38	2.15	2.82	2.68	0.055	0.033	0.549	0.005	0.001	<0.001
**C18:3n-3**	0.32	0.32	0.2	0.2	0.014	0.97	0.652	0.004	0.001	<0.001
**C20:1n-9**	0.17	0.17	0.18	0.18	0.004	0.978	0.266	0.388	0.842	0.824
**C20:3n-9**	0.3	0.3	0.39	0.4	0.010	0.768	0.751	0.01	<0.001	<0.001
**C20:4n-6**	17.07	18.57	19.88	20.36	0.300	0.076	0.231	0.004	0.009	<0.001
**C20:5n-3**	0.15	0.13	0.23	0.21	0.012	0.625	0.622	0.032	0.011	0.007
**C22:1n-9**	0.4	0.43	0.29	0.3	0.015	0.549	0.591	0.003	0.002	<0.001
**C22:4n-6**	0.56	0.53	0.92	0.83	0.041	0.674	0.543	0.016	0.001	0.001
**C22:5n-3**	1.57	1.8	1.68	1.73	0.069	0.262	0.918	0.557	0.74	0.678
**C22:6n-3**	0.72	0.71	0.84	0.76	0.023	0.907	0.23	0.145	0.429	0.279
**SFA ^1^**	45.66	44.72	45.92	45.87	0.305	0.321	0.873	0.797	0.157	0.405
**MUFA ^2^**	31.27	30.76	27.13	26.97	0.461	0.64	0.822	0.002	0.002	<0.001
**PUFA ^3^**	23.07	24.51	26.95	27.16	0.376	0.148	0.576	0.001	0.003	<0.001
**UI ^4^**	1.21	1.27	1.32	1.33	0.012	0.108	0.462	0.007	0.047	0.002
**SUMN3 ^5^**	2.76	2.96	2.95	2.9	0.072	0.366	0.779	0.373	0.772	0.752
**SUMN6 ^6^**	20.01	21.25	23.61	23.86	0.332	0.134	0.492	0.001	0.001	<0.001
**N6/N3**	7.4	7.62	8.19	8.3	0.221	0.749	0.714	0.126	0.33	0.446
**SUMC18:1/C18:0**	1.23	1.22	1.02	0.98	0.033	0.85	0.623	0.026	0.005	0.004
**MUFA/SFA**	0.69	0.69	0.59	0.59	0.014	0.922	0.895	0.019	0.007	0.515

^a^ MSE = Mean square error. ^1^ SFA = Saturated fatty acids; Includes: C14:0, C16:0, C17:0 and C18:0. ^2^ MUFA = Monounsaturated fatty acids; Includes: C16:1n-9, C16:1n-7, C17:1. C18:1n-9, C18:1n-7 and C22:1n-9. ^3^ PUFA = Polyunsaturated fatty acids: Includes: C18:2n-6, C18:3n-3, C20:3n-9, C20:3n-6, C20:4n-6, C20:5n-3, C22:4n-6, C22:5n-6, C22:5n-3 and C22:6n-3. ^4^ UI = Conversion index. ^5^ Includes: C18.2n-6, C20:4n-6 and C22:4n-6. ^6^ Includes: C18:3n-3, C20:5n-3 and C22:6n-3.

**Table 8:** Fatty acid composition (g/100 g total fatty acids) of the experimental diet.

Fatty Acids	%
C14:0	0.488
C16:0	18.722
C16:1n-9	0.173
C16:1n7	0.578
C17:0	0.540
C17:1	0.126
C18:0	3.706
C18:1n-9	23.201
C18:1n-7	0.906
C18:2n-6	46.492
C18:3n-3	3.355
C20:1n-9	0.648
C20:4n-6	0.000
C20:5n-3	0.123
C22:1n-9	0.268
C22:5n-3	0.297
C22:6n-3	0.377

**Table 9:** Detailed information (total number and percentage) on sex distribution (total, male and female foetuses) in the pregnancies studied in the current trial.

Sow ID	GD	Distribution of Foetuses				
		T (n)	F (n)	F (%)	M (n)	M (%)
1331	70	5	2	40.0	3	60.0
1335	70	13	5	38.5	8	61.5
1346	70	7	3	42.9	4	57.1
1347	70	3	1	33.3	2	66.7
1418	70	5	3	60.0	2	40.0
1339	90	4	1	25.0	3	75.0
1402	90	11	5	45.5	6	54.5
1411	90	5	2	40.0	3	60.0
1416	90	3	2	66.7	1	33.3
